# Clinical Review of Trigger Finger in Pediatric and Adult Patients: Evaluation and Management Strategies

**DOI:** 10.7759/cureus.91203

**Published:** 2025-08-28

**Authors:** Daniel Villarreal Acha, Zuhair Zaidi, Abbas Mohamed, Victoria Peters, Muaz Wahid, Sameer A Sajjad, Jennifer Kargel

**Affiliations:** 1 Plastic Surgery, University of Texas (UT) Southwestern Medical Center, Dallas, USA; 2 Surgery, Chettinad Hospital and Research Institution, Chennai, IND; 3 Medical School, University of Texas (UT) Southwestern Medical Center, Dallas, USA; 4 Department of Surgery, Edward Via College of Osteopathic Medicine - Louisiana Campus (VCOM-Louisiana), Monroe, USA

**Keywords:** a1 pulley release, corticosteroid injections, pediatric hand disorders, stenosing tenosynovitis, surgical release, trigger finger

## Abstract

Trigger finger (TF), also known as stenosing tenosynovitis, is a condition characterized by pain, locking, and restricted motion of the digits due to narrowing and inflammation of the flexor tendon sheath, most commonly at the A1 pulley. This review examines both adult and pediatric TF (PTF), emphasizing pathophysiology, diagnosis, and evidence-based treatment strategies. In adults, TF is strongly associated with repetitive finger movements, diabetes mellitus, and inflammatory arthritides. Conservative treatments such as nonsteroidal anti-inflammatory drugs (NSAIDs), corticosteroid injections, splinting, and extracorporeal shock wave therapy are available, though effectiveness varies with disease severity and comorbidities. Corticosteroid injections achieve symptom resolution in 70%-90% of non-diabetic patients, though recurrence rates rise with repeated injections. Surgical options (open, percutaneous, ultrasound-guided, and endoscopic) generally exceed 90% success rates with low recurrence. In pediatric patients, TF is uncommon and often linked to systemic or syndromic conditions. Observation and splinting may be effective in 30%-60% of cases, while surgical A1 pulley release provides resolution in 80%-90%, particularly in severe or multi-digit cases. This review highlights novel treatment algorithms for adult TF and PTF and emphasizes the importance of patient-specific management. A detailed understanding of these distinctions is crucial for optimizing outcomes.

## Introduction and background

Trigger finger (TF), or stenosing tenosynovitis, is a common hand condition characterized by painful locking or catching of the affected digit, primarily caused by inflammation and constriction of the flexor tendon sheath at the A1 pulley. While symptoms may sometimes be mild, untreated TF can progress to significant impairment in hand function and overall quality of life.

TF affects approximately 2% to 3% of the population, predominantly women, peaking in incidence during the fifth and sixth decades of life. Risk factors include repetitive manual tasks and systemic conditions such as diabetes mellitus (DM) and rheumatoid arthritis (RA).

Pediatric trigger finger (PTF), though relatively rare, usually emerges during infancy or early childhood. It can occur in isolation or in association with congenital anomalies and systemic disorders, presenting unique clinical and treatment challenges.

**Figure 1:**
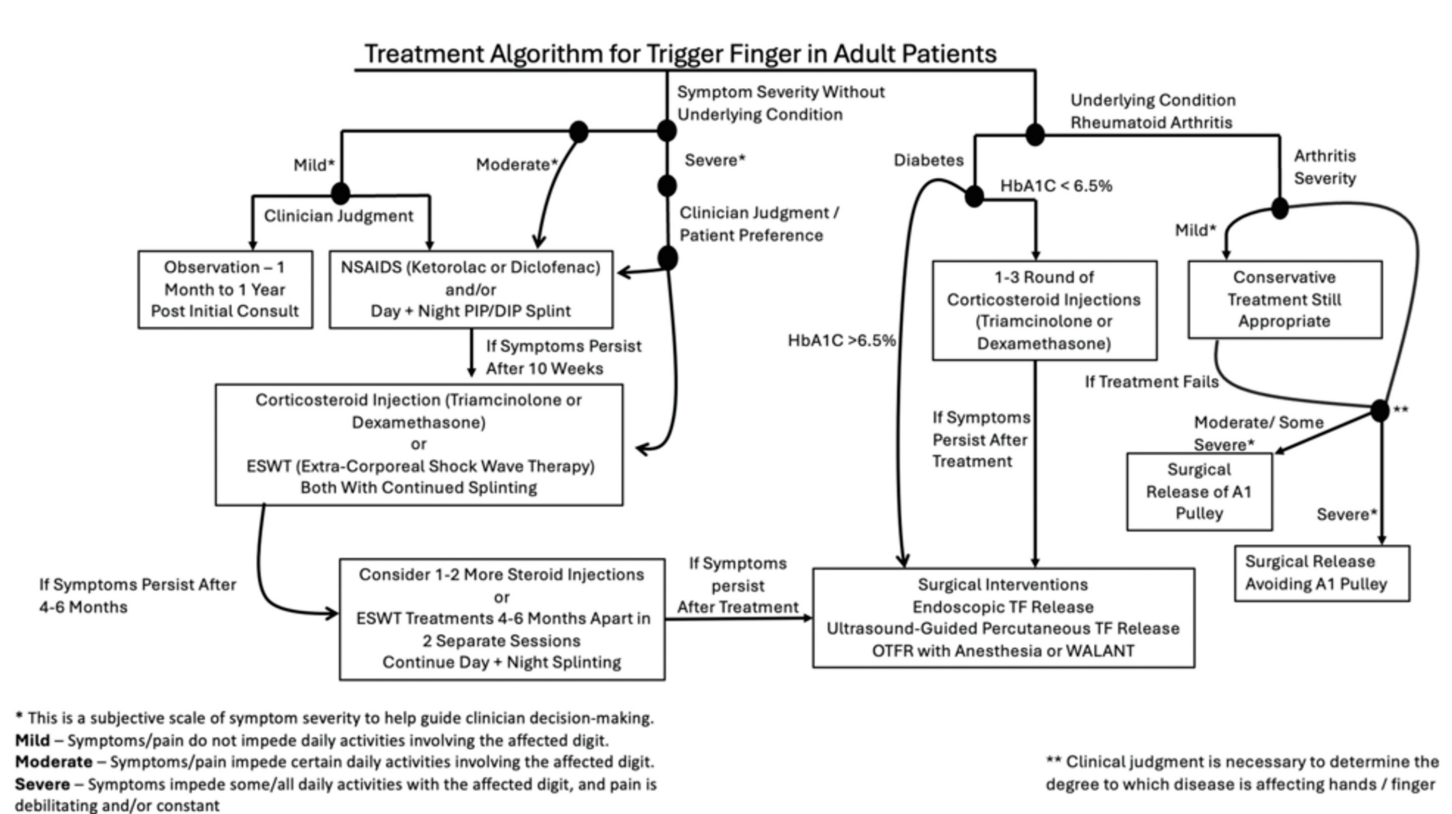
Treatment algorithm for treating trigger finger in adult patients. This figure outlines the treatment approach for trigger finger in adults, starting with the presence or absence of underlying disease, considering symptom severity for those without underlying disease, and then progressing through surgical and non-surgical management options with suggested timelines. This figure was created by the authors and illustrates a proposed treatment strategy for adult trigger finger. No copyrighted material was used. OTFR, open trigger finger release; NSAIDs, nonsteroidal anti-inflammatory drugs; ESWT, extracorporeal shock wave therapy; MCP, metacarpophalangeal; PIP, proximal interphalangeal joint; DIP, distal interphalangeal joint; WALANT, Wide-Awake, Local Anesthesia with No Tourniquet

**Figure 2:**
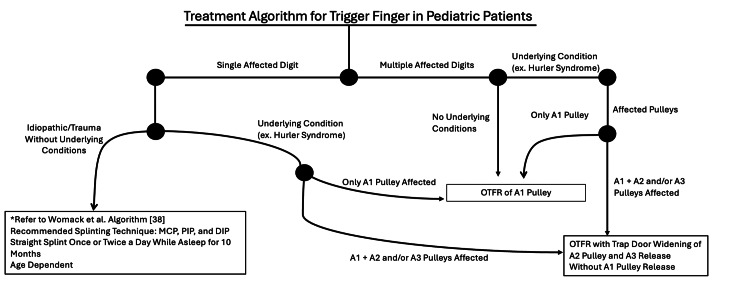
Treatment algorithm for treating trigger finger in pediatric patients. This figure outlines the treatment approach for trigger finger in pediatric patients, starting with the presence or absence of underlying disease and the number of affected digits. The algorithm then progresses through surgical and non-surgical management options. This figure was created by the authors and illustrates a proposed treatment strategy for trigger finger in pediatric patients. No copyrighted material was used. MCP, metacarpophalangeal; PIP, proximal interphalangeal joint; DIP, distal interphalangeal joint; OTFR, open trigger finger release

Treatment options range from conservative measures like nonsteroidal anti-inflammatory drugs (NSAIDs), corticosteroid injections, and splinting to surgical interventions, including open, percutaneous, and endoscopic releases. Effective management requires careful consideration of patient-specific factors, symptom severity, and associated conditions. This review summarizes current knowledge, compares adult and pediatric presentations, and outlines evidence-based treatment algorithms (Figures 1-2) to guide clinical decision-making.

## Review

This narrative review was informed by a structured literature search of PubMed, Embase, and Google Scholar, covering the years 2000-2023. Search terms included "trigger finger," "stenosing tenosynovitis," "pediatric trigger finger," "corticosteroid injection," and "surgical release." Only English language publications were considered. Clinical studies, systematic reviews, and meta-analyses were prioritized, while case reports were included if they described rare pediatric or syndromic presentations. Studies lacking clinical applicability or published without translation were excluded. Although not a formal systematic review, efforts were made to ensure transparency by outlining the search strategy, selection criteria, and limitations of the available evidence.

Adult TF

Epidemiology

TF is a common condition characterized by hand pain and the inability to smoothly flex or extend the affected finger [1]. Epidemiological studies have shown that TF has a lifetime risk of 2.6%, with a higher incidence among individuals with DM and inflammatory arthritides [2]. Women are more affected than men, with a female-to-male ratio of about 3:1 [2]. The ring finger is most frequently affected, followed by the thumb [3]. It can involve one finger, the thumb, or multiple digits simultaneously, with bilateral cases accounting for approximately 10% to 16% of the total [1]. Reports indicate that TF affects up to 20% of individuals with diabetes, with a prevalence range of 4% to 55% for multiple digit involvement, making diabetes a major risk factor for TF development [1,3]. TF commonly occurs during the fifth or sixth decade of life and predominantly affects the dominant hand [1,3]. Additionally, certain occupations that involve prolonged forceful gripping and repetitive finger movements, such as healthcare workers, manual workers, office workers, and musicians, are at an increased risk of developing TF [1]. 

Anatomy

Flexor sheath and synovial membrane: The flexor sheath plays a vital role in housing and guiding the flexor tendons during finger movement. It envelops the flexor digitorum superficialis (FDS) and flexor digitorum profundus (FDP) tendons. Composed of five annular pulleys and three cruciate pulleys, the flexor sheath forms a protective tunnel-like structure [2]. The annular pulleys are numbered A1 to A5 from proximal to distal; they prevent the tendons from deviating laterally or medially and bowstringing on the volar surface of the phalanges. Additionally, the cruciate pulleys, located between adjacent annular pulleys, allow flexibility and facilitate interphalangeal joint flexion [2]. Within the flexor sheath, a thin synovial membrane resides between the sheath and the underlying tendon. This synovial membrane serves to reduce friction during tendon gliding [2]. However, in cases of TF, chronic repetitive friction occurs between the tendon and the A1 pulley. This repetitive friction imparts a high angular load on the underlying tendon during flexion, leading to the development of TF [2].

Histology

Histological analysis of TF reveals several key aspects. Tissue biopsies from patients show fibrocartilaginous metaplasia, with positive staining for S-100 proteins typically found in chondrocytes [2]. Additionally, biopsies of tendons affected by TF display disrupted fibers, hypercellularity, increased chondrocyte presence, and higher levels of glycosaminoglycans. Inflammatory cell or synoviocyte proliferation is not significant in these studies [2]. The digital pulleys, which consist of three layers, demonstrate histologic changes such as progressive destruction of the fibrocartilaginous portion, vascular hyperplasia in the outer zone, fissuring, thinning, and replacement of the fibrocartilaginous gliding surface with fibrous tissue. Neovascular networks form in the outer layer and invade the fibrocartilaginous portion. No inflammatory cells are present in the affected pulley. These histologic findings correlate with the clinical severity of TF/stenosing tenosynovitis [4].

Pathophysiology: Tendon vs. Ligament

While the exact etiology is not fully understood, TF is generally considered a degenerative condition resulting from aging, chronic daily use, and coexisting metabolic disorders [1]. Mechanical factors play a role in the development of TF with repetitive finger movements, repetitive occupational microtrauma, and compressive forces at the A1 pulley among the proposed causes [1,3]. Individuals with metabolic diseases like DM and hypothyroidism have a higher incidence of TF, while conditions such as gout, mucopolysaccharidosis, RA, psoriatic arthritis, and pigmented villonodular synovitis are also associated with its development [1]. Studies have shown an increased occurrence of local disorders like carpal tunnel syndrome (CTS), De Quervain's disease, and Dupuytren's disease in patients with TF. Certain medical conditions, like renal failure, amyloidosis, and sarcoidosis, also pose a higher risk. Although less common, TF can be observed in cases of sesamoid subluxation, abnormalities of the metacarpal head, and tumors [1].

Clinical Diagnosis

The diagnosis of TF is primarily based on clinical findings. The progression of symptoms includes painless clicking, painful triggering, and eventually a flexed, locked digit [5]. Clinicians need to examine all affected digits, as severe symptoms in one finger may mask early signs of triggering in others [1]. Additional symptoms may include tenderness or pain over the A1 pulley, swelling of the metacarpophalangeal (MCP) joint, a palpable, painful tendon nodule, uncomfortable or painful catching with finger motion, thickening of the flexor tendon in the palm, and decreased grip strength [1,3]. Ultrasound examination has shown that the A1 pulley is thicker and stiffer in patients with TF. The degree of thickening seen on ultrasound is correlated with symptom severity [2]. Differential diagnosis may include pyogenic tenosynovitis, palmar fascia thickening, Dupuytren’s contracture, or traumatic injury [3]. The involvement of the pathologic pulley system or the pathologic tendon in TF development remains unclear, as both are likely involved by the time symptoms become noticeable [2]. Various classification systems, such as the Strickland, Boyes, and Green systems, are used to assess the severity of symptoms in TF, but the Quinnell system is more frequently used in clinical and research settings. The Quinnell grading system is a classification method used to assess the severity of TF. It categorizes TF into five grades, ranging from 0 (normal movement) to IV (fixed deformity), based on the degree of finger movement restriction and the ability to actively or passively correct the locking of the digit.

Nonsurgical (Conservative) Interventions

Spontaneous resolution of TF is likely a viable option for treatment before considering more invasive options. One study found that 16% of TFs resolved without treatment within one month of initial specialist consultation [6]. Similarly, another study found that 20% of patients treated with a placebo of normal saline injection within the flexor sheath resolved spontaneously without treatment at a four-month follow-up [6]. More recently, McKee et al. noted a 6% resolution within six to eight weeks in their retrospective analysis of 343 patients with TF, with resolution as high as 90% within one year of initial consultation [7]. 

NSAIDs

Managing TF initially with oral NSAIDs, heat and/or ice, and rest is appropriate [8]. Several studies have provided evidence of the resolution of symptoms with injected NSAIDs. One study comparing Diclofenac and Triamcinolone injections reported a 53% versus 70% resolution in the three-week follow-up, respectively [5]. Another study looking at Ketorolac and Triamcinolone injections reported a higher resolution rate in the Triamcinolone group versus the Ketorolac group in early follow-ups, but at 24 weeks, both resolution rates were comparable, which the authors attributed to spontaneous resolution of TF [5]. 

Corticosteroid Injections

Corticosteroid injections into the inflamed tendon sheath are the first-line treatment when conservative management fails [6]. A single corticosteroid injection can provide relief for up to 10 years for many patients [8]. Additional treatment may involve a second or third corticosteroid injection given four to six months apart, but surgical management is typically considered after two to three unsuccessful attempts [8]. It was found that more than three steroid injections can increase the risk of recurrence after A1 pulley release, suggesting limited benefit in a fourth steroid injection [9]. 

Patients with type 2 diabetes have a lower success rate (66%) with corticosteroid injections compared to those without diabetes (90%) [10]. Sex, TF grade, presence of multiple TFs, and diabetes status did not affect the success of second or third injections [5]. 

A randomized controlled trial found no difference in outcomes between dexamethasone and triamcinolone injections at the three-month follow-up [10]. Seventy percent of trigger digits achieved complete symptom resolution at a mean follow-up of eight years after a single corticosteroid injection. Additional corticosteroid injections may be required for symptom control, with a recommended interval of at least four months between treatments. Steroids can be safely administered up to three times in the same digit before surgery is recommended [10]. 

High-resolution ultrasonography showed improvements in flexor tendon and A1 pulley size after corticosteroid injection, with a decrease in tendon size and at least a single grade improvement [5]. The number of affected digits and the severity of TF influence the response to steroid injections, with multiple affected fingers and higher severity associated with lower odds of symptom resolution. There is no difference in outcomes between subcutaneous and intrasheath corticosteroid injections [5].

Complications of corticosteroid injections include local pain, fat atrophy/necrosis, hypopigmentation at the injection site, and short-term elevation in blood glucose levels for diabetic patients [10]. 

Extracorporeal Shock Wave Therapy

Extracorporeal shock wave therapy (ESWT) has been used for many soft-tissue pathologies such as golfer’s elbow, tennis elbow, rotator cuff tendinopathy, plantar fasciitis, and Achilles tendinopathy. A study comparing cure rate between ESWT and steroid injections in 40 patients with actively correctible TF found no significant differences in cure rate between both treatment choices at one-, three-, and six-month follow-up [8]. This suggests that for patients opposed to undergoing injections or surgical interventions, ESWT may be a viable option. 

Splinting

In addition to steroid injection, splinting is an effective treatment for this condition. The recommended length of splinting treatment ranges from three to 12 weeks, with an average of six weeks [10]. A study demonstrated that overnight MCP splinting in patients with an acute onset (<3 months) TF accomplished complete resolution of symptoms in 55% of patients after their six-week follow-up visit [5]. Another study has provided evidence that proximal interphalangeal (PIP) joint-blocking orthosis leads to more effective reduction in pain, disability, and triggering symptoms than MCP splinting [5].

Surgical Intervention

Open release approach: Open TF release (OTFR) is the preferred surgical technique for treating TF. It involves making a small incision at the base of the affected digit, dividing the A1 pulley responsible for the constriction, and confirming the absence of residual triggering. The procedure is performed using local anesthesia with or without sedation, and patients are encouraged to use their finger while avoiding heavy gripping during the initial recovery period [11]. This approach has the benefit of visualizing at-risk structures, such as the neurovascular bundle, as well as the ability to properly evaluate trigger resolution. A recurrence rate of 2.39% was noted following open TF release [9]. A cohort study of 1,879 patients by Koopman et al. found a complication rate of 17.1% in patients undergoing OTFR, with 14.8% requiring only hand therapy, antibiotics, analgesics, or steroid injections. An additional 2.1% required surgical intervention, and 0.2% developed Complex Regional Pain Syndrome [12]. The preoperative use of lidocaine with epinephrine, recent steroid injection (<80 days), and smoking status are modifiable factors that have been associated with an increased risk of postoperative infection [10]. A study by Kloeters et al. compared three incisions: transverse in the distal palmar crease, transverse and 2mm distal to the distal palmar crease, and longitudinal over the MCP joint without crossing the distal palmar crease. They noted that the longitudinal incision resulted in the least scarring and the fastest DASH score reduction [13]. The Wide-Awake, Local Anesthesia with No Tourniquet (WALANT) procedure has risen in popularity due to its high patient satisfaction rate, decreased postoperative downtime, and cost-effectiveness [13]. 

Percutaneous Approach

Percutaneous TF release is a minimally invasive alternative to open release. In the past, this option was done blindly with a 16- to 18-gauge needle, relying on the clinician’s ability to palpate important anatomical landmarks, and was associated with unintentional A-2 release, causing *bowstringing*. In a 2013 study by Guler et al. comparing surgical and percutaneous release techniques, iatrogenic digital nerve injury was noted in 5.7% of the percutaneous release group, and, although statistically insignificant, caused the authors to proclaim open release as the safer alternative. Now, new techniques using ultrasound-guided 16-gauge or bladed-tip 18-gauge needles have surfaced, decreasing the risk of complications [14-16]. In past years, the percutaneous technique without ultrasound had a reported long-term success rate of 87% [17]. With the addition of ultrasound guidance, that success rate has risen to 91%-100% [16,18]. Another study compared open versus ultrasound-guided percutaneous approaches and found that pain at one-month follow-up was significantly less in the percutaneous group, with a patient satisfaction rate of 76.4% to 94.1%, respectively [19]. 

Endoscopic Approach

Through endoscopic approaches, scarring and healing time can be kept at a minimum, while maintaining proper visualization for a non-inferior risk of injury to important structures [8]. One study compared Open versus Endoscopic TF release (ETFR) and found major complications to be unlikely and minor complications to be similar in both groups, 8% versus 6%, respectively. ETFR has been shown to increase postoperative mobility, decrease discomfort for patients, and lead to a quicker return to work and daily activities [20].

Special Considerations

Diabetes: TF occurs at a significantly higher rate of 20% in the diabetic population [10]. It is known that the pathophysiology of DM leads to impaired healing and a higher propensity for infection. A meta-analysis by Atthakomol et al. reported that the risk of infection was 65% higher in diabetic patients compared to non-diabetic patients [21]. A study by Gundlach et al. compared diabetic versus non-diabetic patients’ complication rates from undergoing carpal tunnel release and/or TF release. They noted an increased risk of wound healing complications in the diabetic group, particularly when HbA1c exceeded 6.5% [22]. Similarly, another study noted that an HbA1c value above 6.5%, ipsilateral concomitant hand disease, or symptom presence for greater than 2.5 months, increases the risk for failure of local corticosteroid injection [23]. Infection risk has also been quantified as 65% higher in patients with diabetes after open TF release compared to those without diabetes in patients of all ages and those in both non-obese and obese groups [21]. A recent study by Hollins et al. looked at the differences in outcomes between patients with type 1 DM (T1DM) and type 2 DM (T2DM) undergoing OTFR. They found that women with T1DM reported significantly worse stiffness, while women with T2DM reported worse pain with load, pain during motion without load, and overall worse results at three months. However, no significant differences were noted at 12 months. Of note, women with T2DM had a higher Quick Disabilities of the Arm, Shoulder, and Hand (QuickDASH) score at three and 12 months [24]. Corticosteroid injections in diabetic patients used to be reported as less effective than injections in non-diabetic patients [5]. However, more recent studies have found no significant difference in initial, second, or third corticosteroid injection efficacy in diabetic patients [5]. Transient hyperglycemia is a risk associated with corticosteroid use in diabetic patients. The effect is short-lived, however, and does not outweigh the benefits associated with this treatment. 

Rheumatoid arthritis:* *RA is a chronic inflammatory polyarthropathy characterized by autoantibodies against the synovium, leading to intra-articular inflammation, degradation of cartilage, ligaments, and bone. One of the first manifestations of RA may be trigger digit [6,25]. It is not uncommon to see multiple affected digits along with flexor tendon entrapment [6]. Before the introduction of modern medical therapy (Disease-Modifying Antirheumatic Drugs (DMARDs)), the literature strongly advocated for preservation of the A1 pulley in favor of tenosynovectomy and/or FDS slip excision when treating trigger digits in patients with RA. Chronic inflammation in these patients caused tenosynovial thickening, collateral ligament laxity, and destabilization of the MCP joint. Release of the A1 pulley in these patients was therefore thought to accelerate ulnar deviation and volar subluxation. In the last three decades, however, therapies like DMARDs and tumor necrosis factor-alpha (TNF-α) inhibitors have surfaced to delay disease progression and thus decrease the prevalence of severe joint deformity. Treatment with corticosteroids has been proven to be effective in patients with RA, relieving pain and triggering/locking by five days and three weeks, respectively [6]. Treatment of TF in patients with RA is nuanced and evolving. Clinician judgment is imperative to provide the best result for these patients, considering disease progression and medical treatments available, as treatment algorithms are constantly evolving for this condition.

Blood thinners: For those patients who undergo any surgery, the benefits of cessation of antithrombotic therapy should be weighed against the drawbacks, such as increased risk of thrombotic events like stroke, pulmonary embolism, or coronary events. The use of antithrombotic therapy in patients who undergo TF release or similar operations has been studied. In a systematic review by Sardenberg et al., it was found that major complications (defined as bleeding or bruising requiring surgical intervention) were quite uncommon (0.7%) and only seen in more complex and involved hand surgeries like proximal row carpectomy or wrist arthrodesis. Minor complications like bleeding or bruising that did not require surgical intervention were more common (8.5%).

Pediatric TF

Epidemiology and Clinical Diagnosis

PTF is a relatively rare condition that typically develops within the first year of life. Studies have indicated that PTF is less common than trigger thumb in infants and children, with an estimated prevalence 10 times lower [26]. No predilection for sex has been noted [27]. In a prospective study involving 7700 neonates, no cases of congenital TF were found, while a retrospective study identified eight cases that were diagnosed and resolved within the first year of life [28]. This suggests that PTF tends to emerge during early infancy. PTF can affect any finger, but it predominantly affects the long finger, accounting for nearly 50% of cases, with the Index being the least common digit affected [27,29]. Physical examination typically reveals diminished active range of motion, triggering, and/or fixed flexion contracture. Additionally, a palpable volar mass over the MCP joint, caused by nodularity in the FDS or FDP, is often present [26]. MRI can help visualize these abnormalities.

Pathology, Pathophysiology, and Etiology

The pathophysiology of PTF is poorly understood but has shown an association with several other concurrent medical conditions, such as DM, juvenile RA, trisomy 18, Ehlers-Danlos syndrome, central nervous system (CNS) disorders, and mucopolysaccharide storage disorders like Hunter or Hurler syndrome [29]. In a systematic review by Wong et al., 29% of PTF cases were associated with an underlying condition [30]. Other associations include intratendinous calcifications, granulation tissue trauma, and anatomical anomalies such as abnormal lumbricals or decussation of the FDS tendon proximal to the A1 pulley [26,29,30-33].

Nonsurgical (Conservative) Interventions

The most common conservative interventions for PTF include nighttime extension splinting, stretching, NSAIDs, and corticosteroid injections. However, splinting and observation are performed in less than 10% of cases and lack studies defining treatment outcomes and data on symptom relief rates [15]. Similarly, as of July 2023, there are no studies quantifying the effectiveness of steroid injections for the treatment of TF in the pediatric patient population. A retrospective cohort study by Jia et al. provided a rate of complete resolution of PTF with conservative treatment (81/270, 30.0%), with 75% of them having symptom resolution by six months and 89.1% by 12 months. In addition, a recent systematic review by Womack et al. evaluated seven articles and 118 TFs, determining that those fingers treated non-surgically, with either steroid injections or splinting, had a resolution rate of 57.8% (37/64) as opposed to those treated surgically having an 87% (47/54) resolution rate.

Splinting has the benefit of placing the tendon in an extended position, letting the affected tendon rest [8]. However, little or contradictory evidence is available on the effectiveness of splinting in pediatric patients. In a comparative study by Shiozawa et al., pediatric patients (mean age two years) were fitted with splints on fingers with the wrist, MCP, PIP, and distal interphalangeal (DIP) joint extended to neutral. The splints were worn once or twice a day while the child slept during naptime and/or the first 2 or 3 hours of sleep at night. It was found that 67% of patients with TF treated had symptom resolution compared to 30% in the observation group, with an average time to symptom resolution of four years 11 months [34-35]. However, in their retrospective cohort study, Jia et al. noted that the resolution rate with splinting was not clinically significant compared to no treatment (30.8% vs. 29.4%, respectively). Additionally, complete resolution with splinting became significantly less likely as the grade of the PTF increased from 1 to 2-4 (via the Quinnell grading system) [27]. Jia et al. also compared results from Shiozawa et al. and concluded that younger patients (2 vs. 5.5 years) splinted for longer (10 months vs. 4.8 months) may have improved treatment outcomes [27]. This conclusion warrants further investigation.

Surgical Interventions

The gold standard procedure for treatment of PTF is open A1 pulley release. Studies have reported residual flexion contracture of the IP joint with delayed surgical management of patients older than three years of age [33]. Although timeliness in surgical treatment is essential, to fully eliminate triggering, further procedures may be necessary. Studies have proposed an extensile incision to properly release the A1 pulley and adequately visualize the FDP and FDS tendons, examining for decussation and other anomalies. As pediatric cases are often more complex than their adult counterpart, further dissection of structures like the A2, A3 pulleys, or FDS tendon may be necessary [35]. With an open approach, general anesthesia will be necessary; thus, a full medical evaluation of the patient is warranted to prevent any adverse reactions in the operating room or post-op. Once the wounds have healed, hand therapy may be necessary to prevent joint contracture and muscle wasting, via passive stretching exercises and active motion therapy. If symptoms persist, additional surgical exploration and synovial biopsy may be necessary, along with serological markers for possible Juvenile RA [36].

Approaches and Special Considerations

Jokuszies et al. surgically treated six children with Hurler syndrome (a mucopolysaccharidosis) and TF and observed not only the A1 pulley being affected but a higher rate of A2 and A3 pulley involvement. Out of 43 fingers affected, seven had A1 pulley involvement, whereas 27 and 25 had A2 and A3 involvement, respectively. When all three of these pulleys were involved, a trap door widening of the A2 pulley and release of the A3 pulley was performed to avoid weakening of the tendons, with self-reported very good results and symptom-free patients [37]. Due to the etiology of the disease, CTS is also prevalent in these children, and if both TF and CTS are present, concurrent surgical management may be required. 

Proposed Treatment Regimen for TF in the Adult Patient

In a concept paper by Gil et al., a treatment algorithm was proposed describing both conservative and surgical approaches to the treatment of adult TF [5]. This algorithm was updated with current advances in the field and displayed in Figure 1.

Proposed Treatment Regimen for TF in the Pediatric Patient

In a systematic review by Wong et al., it was found that multiple trigger digits had a correlation with failure of conservative treatment. With this and previous information, a treatment algorithm was proposed: If multiple affected fingers are present and an underlying condition in present, proceed with appropriate workup/referrals and treat surgically; if only one finger is affected and/or history of trauma to the finger is noted, a trial of conservative treatment is recommended and if that fails then a surgical approach is appropriate [30]. 

A recent systematic review by Womack et al. evaluated seven articles and 118 TFs, determining that 50% (21/40) of fingers had resolution of triggering with observation alone, 66.6% (12/16) resolved with splinting, and 87% (47/54) resolved with surgical intervention. They proposed a treatment algorithm that included a breakdown of observation, splinting, and specific surgical options if conservative methods failed [38]. 

A treatment algorithm was built based on these studies, along with current advances in the treatment of PTF, and is displayed in Figure 2.

## Conclusions

TF is common in adults, particularly in those with diabetes and inflammatory arthritides, while pediatric cases remain less common. In adults, corticosteroid injections are the first-line therapy, with surgery reserved for refractory disease; in children, surgery is often required due to limited success with conservative measures. Key research gaps include the absence of pediatric steroid studies and the need for long-term comparative trials of ESWT and other minimally invasive approaches.
